# Home stimulation, development, and nutritional status of children under 2 years of age in the highlands of Madagascar

**DOI:** 10.1186/s41043-023-00399-x

**Published:** 2023-06-29

**Authors:** Hasina Rakotomanana, Deana Hildebrand, Gail E. Gates, David G. Thomas, Fanjaniaina Fawbush, Barbara J. Stoecker

**Affiliations:** 1 Department of Nutritional Sciences, Oklahoma State University, 301 Nancy Randolph Davis Building, Stillwater, OK 74078 USA; 2 Department of Psychology, Oklahoma State University, Stillwater, USA; 3 Department of Agricultural and Food Science and Technology, University of Antananarivo, Antananarivo, Madagascar

**Keywords:** Home stimulation, Child development, Undernutrition, Madagascar

## Abstract

**Background:**

The Vakinankaratra region of Madagascar has a high burden of child undernutrition which, in addition to poor psychosocial stimulation, is a strong risk factor of poor child development. However, there are limited studies evaluating the relations between developmental deficits, child nutrition outcomes, and home stimulation in the region. The purpose of this study was to assess the development of children aged 11–13 months in relation to their nutritional status and to examine parental home stimulation attitudes and practices in the Vakinankaratra region.

**Methods:**

Cognitive (*n* = 36), language (*n* = 36), motor (*n* = 36), and socioemotional (*n* = 76) development were assessed using the Bayley Scales of Infant and Toddler Development III. Household stimulation environment was evaluated using the family care indicators survey. Stunting (length-for-age *z*-score < − 2) and underweight (weight-for-age *z*-score < − 2) were determined using the 2006 WHO growth standards. Perceptions of and barriers to greater home stimulation for children were collected using focus group discussions among parents and in-depth interviews with community nutrition agents.

**Results:**

Almost all mothers reported that parent–child interaction with talk and play was very important. Alarmingly high stunting rates (> 69%) were observed in this subsample. Limited time and tiredness were the major barriers to home stimulation mentioned by parents and confirmed by key informants. Children had a very limited variety of play materials, and most of the mothers used household objects (75%) and materials from outside the house (71%) as children’s toys. Composite cognitive [mean (SD): 60 (10.3)], motor [61.9 (13.4)], language [62 (13.2)], and socioemotional [85.1 (17.9]) scores were low. Fine motor, cognitive, and receptive and expressive language scores were correlated [0.4 < *r* < 0.7, *p* < 0.05].

**Conclusions:**

The very high stunting rates and very low performance on cognitive, motor, language, and socioemotional development assessments of children in the Vakinankaratra region require urgent attention.

## Background

The first 2 years of life have been recognized as a critical window of opportunity for infants and young children based on the large body of evidence that shows that several of the biological processes required for appropriate growth and development occur during that period [1, 2]. Children having poor development are less likely to reach their full potential and will be less productive as adults [3]. Inadequate development during childhood has been associated with fewer years of schooling and lower academic achievements, ultimately leading to lower earnings in adulthood [3]. Child development is heavily influenced by genetic, environmental, and nutritional factors starting immediately after conception [4]. Appropriate nutrition is fundamental for optimal brain formation [2, 5–8], and linear growth faltering has been identified as a strong risk factor for poor development because of its consistent association with developmental delays [3, 9–11]. Additionally, there is some evidence that early childhood infection is associated with lower cognitive scores later in life [12, 13]. Repeated episodes of diarrhea also can lead to stunting and can increase the risk for impaired development [9]. However, diarrhea may negatively impact child cognition independently of stunting [14]. Similarly critical are parent–child interactions that provide the child adequate learning opportunities [15, 16]. These include supportive learning environments with appropriately stimulating toys and other educational materials to which the child has access [17, 18]. Adequate cognitive stimulation during infancy has been consistently associated with higher cognitive scores later in childhood [15, 18], and gains are likely sustained in adulthood [19].

Two-thirds of the estimated 250 million children affected by suboptimal development live in sub-Saharan Africa [20]. In Madagascar, two of the strongest risk factors for impaired development, child stunting and extreme poverty [3, 15], are widespread, and delayed development has been reported for Malagasy children [21, 22]. There is also evidence of disparities in child developmental outcomes across socioeconomic status and across regions of residence [21, 23]. The Vakinankaratra region has the highest child stunting prevalence in Madagascar previously reported at 60% [23], making the majority of the children at risk of not reaching their full potential. In addition, although widely recognized as a critical component promoting mental development, psychosocial home stimulation for Malagasy infants and young children has rarely been investigated. Moreover, there are limited data on the interplay among nutrition, psychosocial home stimulation attitudes and practices, and developmental outcomes in this region. Such data are key to provide local context to inform interventions and policies aiming to ultimately improve child development in similar settings as the Vakinankaratra region.

## Methods

The purpose of this study was to assess the development of 11–13 months children and parents’ attitudes toward and practices of stimulation in their homes in the Vakinankaratra region of Madagascar. This study sought to (1) evaluate home stimulation practices for infants and young children; (2) assess parental perceptions and barriers to home stimulation; (3) describe the development of 11–13-month-old children; and (4) determine associations among developmental domains in the sample. The subsample for this study was drawn from a larger study of 391 6–23-month-old children investigated to identify factors influencing child undernutrition in two rural districts of the Vakinankaratra region (Antanifotsy and Antsirabe II) located in the central highlands of Madagascar. Details of the larger study are described elsewhere [24]. Child development assessment for this study was conducted on a subsample of all children 11–13 months of age.

### Home stimulation practices

The family care indicators (FCI) survey [25, 26] was used to assess family care practices indicating the available support for child stimulation in each household. The FCI are commonly integrated into nationally representative surveys to assess risks and protective factors of child development. Two source of play materials and variety of play materials subscales, the most strongly predictive of child development in Bangladesh [26], were adapted to the local context and added into the survey questionnaire to collect information regarding home stimulation activities and materials. Yes or no questions were asked of the mothers on play activities that adults in the household performed with the child, as well as on sources and varieties of play materials. FCI items deemed not relevant for the local context or to which mothers were most likely to respond “no,” for example use of children’s picture books and toys for drawing or writing, were not added the questionnaire. Means and SD were calculated for the two subscales, and frequencies were aggregated for other FCI indicators included in the survey to give insights on home stimulation practices and activities in rural Madagascar.

### Child development assessment

The Bayley Scales of Infant and Toddler Development (Bayley-III) [27] were used to assess cognitive, language, motor, and socioemotional development on 11–13-month-old children. Culturally inappropriate illustrations in the picture book were replaced with more relevant pictures. For example, an adult vacuuming was replaced by a mother sweeping the floor with a broom, and pictures of Caucasian and Asian children were replaced with pictures of Malagasy children. Based on child development courses and mentoring on child assessments by a psychologist (DGT), the cognitive, language, and motor scales were all assessed for a subsample of 36 toddlers by the lead author (HR) at the local community nutrition sites after pre-testing on two Malagasy children aged 13 and 15 months not included in the study. Testing began for each child with tasks designated for the preceding age group (9 months, 0 days–10 months, 30 days), and children had to have successes on three consecutive items in order to continue. For each scale the ceiling was reached when a child did not succeed on five consecutive items. Children received a score of “1” on each item if they showed the expected behavior and “0” otherwise. The sum of successful tasks was added to the number of tasks below the point where testing began in order to obtain the raw scores.

Socioemotional development was assessed using Likert-scale questions asked of the mothers of all 76 children aged 11–13 months in the original sample. Questions were translated into Malagasy and were then reviewed by two mothers and two fathers of children younger than 2 years who were also native speakers. Interviewers were trained to ask probing questions as necessary to help the mothers better understand the concepts being asked. Raw scores were obtained by summing all the responses. Assessments were conducted by the lead author (HR), a doctoral student in nutritional sciences at the time of data collection. HR took courses in early child development and was mentored by a psychologist (DGT) on child development assessments. HR conducted Bayley assessments on two children aged 13 months outside of the study sample before data collection.

Raw, scaled, and composite scores for each subscale of child development were calculated as specified in the Bayley-III manual [27] and reported for each domain and subdomain. Scaled scores, calculated from raw scores, and adjusted for age, have a range from 1 to 19 with a mean of 10 and a standard deviation of 3 for each subdomain. Composite scores in the Bayley Scales are also age-adjusted but at the domain level (cognitive, motor, language, and socio-emotional) and are set to range from 40 to 160 with a mean of 100 and a standard deviation of 15. Scaled and composite scores are used to compare the performances of children at equivalent ages.

### Anthropometric status

Child recumbent length was measured to the nearest 0.1 cm using a wooden length board. Two measurements were taken, and the mean was taken. Child weight was measured using a hanging scale (SECA). Child length and weight were converted to length-for-age (LAZ), weight-for-age (WAZ), and weight-for-length (WLZ) *z*-scores using the 2006 WHO Growth Standards [28]. A *z*-score < − 2 indicated stunting for LAZ, underweight for WAZ, and wasting for WLZ.

### Statistical analyses of quantitative data

Descriptive statistics were used to report the characteristics of the study population as well as FCI indicators, child development scores, and growth outcomes. Pearson’s correlation was used to identify relations between developmental domains. Analyses were conducted using R (R Core Team, Vienna, Austria), and statistical significance was set at *p* < 0.05.

### Qualitative analysis of perceptions and barriers to greater child home stimulation

A qualitative approach was used to collect and analyze data on the perceptions and barriers to greater child stimulation (Table 1). A total of 10 focus group discussions (FGDs) among mothers (*n* = 46) and fathers (*n* = 17) of children aged 6–23 months were conducted. Key informant interviews (KII, *n* = 8) were also held individually with community nutrition agents (CNAs), a nurse and a non-governmental organization (NGO) field monitor. The lead author led the discussions in the local language (Malagasy) and took field notes. Gathering information from various sources (CNAs, mothers, and fathers) using different methods (focus groups and interviews) allowed for triangulation and increased the trustworthiness of the qualitative data. During data collection, saturation was deemed to be reached when no new information was obtained during the FGDs or KIIs. Discussions were audio-recorded, transcribed verbatim, and translated into English before analysis. Next, the lead author familiarized himself with the transcripts and the field notes. Patterns found in the data were grouped into codes addressing the research purpose in assessing parental perceptions of home stimulation practices and in identifying barriers to greater home stimulation. Themes identified were discussed and agreed with co-authors.

**Table 1:** Focus group discussions and interview guide

*Focus group discussion mothers*
1. What do you do when you spend time with your child?
2. How important is it to you to find time to play/talk to your child?
3. If you wanted to play and talk to your child more, what prevents you from doing so?
*Focus group discussion fathers*
1. How important is it for you to find time to play/talk to your child?
2. If you wanted to play and talk to your child more, what prevents you from doing so?
*Individual in-depth interview*
1. How important is it for mothers to find time to play/talk to their child?
2. How important is it for fathers to find time to play/talk to their child?

## Results

Data for children assessed for social–emotional development (*n* = 76) and for the subsample tested for cognitive, language, and motor development (*n* = 36) were similar (Table 2). Essentially, half of the children were females, and low birthweight was > 20% in both subgroups. Among the 76 children evaluated for socioemotional development, 72% were stunted, 21% were underweight, and three suffered from wasting (4%). Two-thirds of the children evaluated for cognitive, language, and motor development were stunted and 11% were underweight. Mean maternal age was similar, and almost all mothers (97.4%) had at least some primary education. Mean (SD) household size was 4.3 (1.3), and almost half of the households did not use iodized salt.

**Table 2:** Characteristics of the study population

Variables	Social–emotional development assessment *N* (%)	Cognitive, motor, and language assessment *N* (%)
*Child characteristics*		
Age (months)		
11	33/76 (43%)	13/36 (36%)
12	29/76 (38%)	12/36 (33%)
13	14/76 (19%)	11/36 (31%)
Sex		
Male	37/76 (49%)	18/36 (50%)
Female	39/76 (51%)	18/36 (50%)
Birthweight		
< 2500 g	47/76 (21%)	10/36 (28%)
≥ 2500 g	29/76 (79%)	26/36 (72%)
Nutritional status		
Stunting	55/76 (72%)	25/36 (69%)
Underweight	16/76 (21%)	11/36 (11%)
Wasting	3/76 (4%)	0/36 (0%)
*Maternal characteristics*		
Age, mean (SD)	27 (6.5)	27 (7)
Education		
No formal education	2/76 (3%)	1/36 (3%)
At least some primary	40/76 (53%)	17/36 (47%)
At least some secondary	30/76 (39%)	14/36 (39%)
Higher	4/76 (5%)	4/36 (11%)
*Household characteristics*		
Household size, mean (SD)	4 (1.4)	4 (1.3)
Use of iodized salt		
No	36/76 (48%)	17/36 (47%)
Yes	39/76 (52%)	19/36 (53%)

### Home stimulation practices

**Fig. 1:**
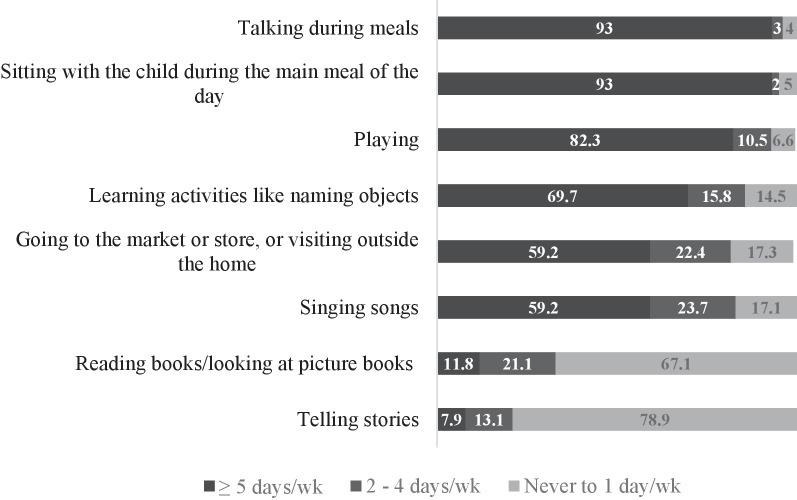
Frequencies of play activities of young children aged 11–13 months (%, *n* = 36)

Household items, such as kitchen utensils (75%), and materials from outside the house, such as wooden sticks (71%), were common children’s toys (Table 3); otherwise, children had a very limited variety of play materials (Table 3). More elaborate toys such as those made for building or constructing (8%) and toys for pretending (26%) were less common. Most (> 80%) of the mothers reported that an adult was talking to their infants, sat with their infants during meals, or played with their infants almost every day (Fig. 1), but telling stories or reading books were not common activities.

**Table 3:** Selected items from the family care indicator subscales for young children aged 11–13 months (%, *n* = 36)

Item	Frequency (%)
*Source of play materials*	
Household objects	75
Materials from the house compound	71
Toys made by an adult	39
Mean (± SD)	1.78 (1.04)
*Variety of play materials*	
Toys that make noise/music	58
Toys for pretending like dolls	26
Toys for building/constructing	8
Mean (± SD)	0.92 (0.77)

### Child development

Although the Bayley-III has been validated for use in low- and middle-income countries around the world, to the best of our knowledge it has not been specifically validated in Madagascar. Our assessment for each scale began as recommended with the tasks for the age category immediately below the age of the children (i.e., at 9–11 months); nevertheless, two infants (5%) failed to surpass the expected starting point (11–13 months) for their age for the general motor skills. Furthermore, for cognition, fine motor, and expressive language subdomains, only 10 infants out of the 36 succeeded in passing the expected starting point for their age (72% failed). Scaled child development scores were low across all domains (Table 4). Likewise, children performed poorly across the cognition [60 (10.3)], motor [61.9 (13.4)], and language [62 (13.2)] domains. However social–emotional composite development scores were higher [85.1 (17.9)] than the other domains.

**Table 4:** Mean (SD) Bayley scales development scores for each domain

Domain/subdomain	*N*	Raw score	Scaled score (0–19)	Composite score (40–160)
Cognitive	36	26 (4)	2 (2)	60 (10)
Gross motor	36	31 (6)	4 (2)	–
Fine motor	36	21 (4)	3 (3)	–
Motor domain	36	–	7 (4)	61.9 (13)
Receptive language	36	9 (4)	5 (3)	–
Expressive language	36	2 (2)	2 (2)	–
Language domain	36	–	7 (4)	62 (13)
Socioemotional	76	64 (11)	7 (4)	85 (18)

**Fig. 2:**
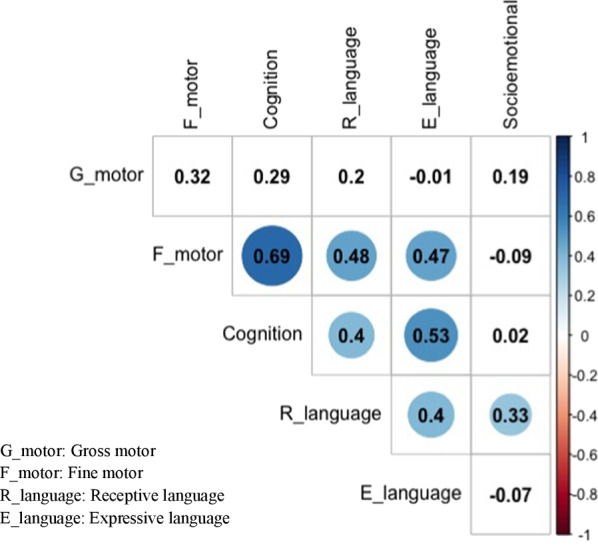
Correlation coefficients between the child development scores per domain (*n* = 36). Correlation coefficients within circles are statistically significant (*p* < 0.05)

The strongest correlation was between cognitive and fine motor subdomains (*r* = 0.69, *p* < 005). Cognition was also correlated with both of the language subdomains (Fig. 2). Language subdomains were correlated significantly with each other and with the fine motor skills. The significant correlation coefficients ranged from 0.33 to 0.69. Only the gross motor subdomain did not correlate significantly with other subdomains.

### Perceptions and barriers to greater child home stimulation

Almost all mothers in FGDs mentioned that spending time with their children was important. Mothers reported that children will become smarter and will have better language skills if parents interact with their children often.

“Their [young children] minds will expand when you [mother] talk to them” Mother, FGD 3.

“Because it happens when the mother doesn’t talk to the baby, then they won’t talk either, or it will take a long time. Even they are very curious when you start talking or teaching them to talk. When they are born, it comes naturally, only few mothers don’t talk to their babies” Mother, FGD 6.

Another reason mentioned by a few mothers regarding the importance of spending time with children was that the stronger mother–child bond that will make the child happy.

“[Spending time with the child] bonds the mother and the child” Mother, FGD 4.

Most mothers in the FGDs reported playing and entertaining their children as well as talking to them as the most common interaction. Playing included making the child dance or grab a toy and singing.

“We [mothers] build for them [children] small cars and they push them around” Mother, FGD 1.

“For example, ask them to grab a cup or a container, a spoon, and ask them to place it somewhere else, and they know” Mother, FGD 2.

A few mothers also considered doing household chores such as cooking and cleaning while holding the child as interaction.

Similarly, all fathers in the FGDs also mentioned that spending time with their children was important, although the reasons were slightly different than those expressed by the mothers. Fathers in two FGDs reported that spending time with their children will strengthen the father–child bond and will be better for the family as a whole. A father stated that it will also prevent some men from being unfaithful and drinking alcohol.

“Yes, it is important… Because it has serious consequences for his life” Father, FGD 1

“You are also happy to play with your child because in the evening you won’t go out, thinking of something else like drinking, or even having a girlfriend because you don’t have anything to play with, so you’d go and find something else to do” Father FGD 3

A few fathers mentioned that children will become shy and slow if they do not spend time with them.

“…even the youngest you should talk to him too so that he doesn’t become mute” Father, FGD 3

Key informants confirmed the parents’ perceptions regarding the importance of interacting with the children.

“From the way I see it, it is important for them because when they see other people’s children who are doing well, they are thinking that it is just normal to talk and play with their children and that they should do that” KII 3.

“Yes, they really know it’s important because that’s what we teach them here” KII 1.

Limited time and tiredness were the major barriers mentioned by fathers and mothers preventing them from spending more time interacting with their children. Mothers stated that they do not have time because of work and household chores, while a few fathers mentioned many men in the community leave their families for work for long periods of time. Key informants gave similar answers confirming that parents have limited time due to their work and chores and caring for their other children.

“We [mothers] don’t have a lot of time to play [with the children]” Mother, FGD 3.

“Because you don’t have time to play with your child for half a day, you have to work and look for money. So, one or two hours at most then you’d have to go find other things to do” Father, FGD 3.

“Yes, it is important, they know it’s important…But the thing is they [mothers] don’t have time” KII 4.

“Besides the time, I also think if the father has some work, then he doesn’t have time to play with his children” KII 2.

One key informant mentioned that for some fathers, working is more important than spending time with their children. Several mothers and fathers stated that they are tired from work in the evening and may not have the time to interact with their children. A few fathers reported that they do not spend time with their children when they are dealing with a problem or when they have marital disputes.

“Yes, they [fathers] only think about work” KII 2.

“For me when I am tired from [working in] the fields” Mother, FGD 4.

“One thing that can happen is that there might be a quarrel between the husband and wife, so when they are angry or upset, they won’t care about the baby anymore, and one of them will go away” Father, FGD 4.

## Discussion

Children in our sample had low cognitive, motor, and language development scores, which exceeded 2 SD below the mean of standardized scores for children of the same age. These scores were also lower than reports from other LMICs [29, 30]. Because we are unaware of published validations of the Bailey-III in Madagascar, conversion of these scores to expected values for age is problematic. Also, our sample may not be representative of the entire Vakinankaratra region because of it is a subsample from a larger study and we only assessed children 11–13 month old; however, these results do provide a view of the extent of the child development deficits in rural areas of Madagascar.

Stunting was identified in 72% of all children in our study and 21% were underweight. Although this study was not designed to detect differences in child development across nutritional status, we observed a pattern showing lower cognition and language scores among stunted and underweight children compared to children with optimal growth. Stunting, reflecting chronic malnutrition, has been consistently associated with lower cognitive scores and reduced school achievements later in childhood [3, 9, 10, 12, 31]. In addition, positive linear association has been reported between length/height and development in Tanzanian children, suggesting that shorter children are more likely to experience developmental deficits regardless of their stunting status [11]. The very low mean LAZ (− 2.50) among children 6–23 months in the Vakinankaratra region [24], suggests that most infants are shorter than their age norm and thus are at high risk of impaired development.

Most of the children’s toys were items from the household or objects from the outdoors, none of which were specifically designed for child stimulation. Children also had a limited variety of play materials, mainly toys that make noise. A wide variety of toys has been shown to be strongly associated with improved child development [10, 32]. Increasing accessibility to various play materials that increase child stimulation such as picture books, toys for learning shapes, and toys for pretending would likely benefit the children. 

The parents’ reported positive attitude toward child stimulation can be an opportunity to promote child development. Although fathers and mothers have different motives, they recognize the importance of spending time with young children. Mothers appeared to be more aware of the links between parent–child interaction and child development than fathers. Parents able to make the link between interactions with their child and the child’s development are more likely to engage in practices that promote stimulation [33]. Fathers could benefit from sensitization regarding the connection between positive stimulation and their children’s development and the same message could be reinforced among mothers.

In most households, adults reported engaging in play activities with their children on most days (more than 5 days/week). A variety of adult–infant interaction has been associated with better stimulation practices and subsequent improved developmental outcomes in children [10]. Parents and other caregivers in the family need to be encouraged to diversify the type of activities they participate in with the child every day.

In addition, to successfully promote home stimulation for children, the identified barriers need to be addressed. In most families in the rural areas of the highlands of Madagascar, both parents usually work, and they have limited time for longer child–parent interactions [34]. Providing parents suggestions for quick, practical, and easy to implement activities that promote child development may be needed as lack of time was the major barrier. Such activities, which should be both culturally appropriate and adequate for the infants’ developmental stage, may be identified with further research based on activities in which parents are already engaging. Moreover, encouraging the involvement of other members of the household, including grandmothers, in providing stimulation to young children may be helpful. Grandmothers’ involvement in child care has been associated with better child fine motor and cognitive skills [35, 36].

The findings that cognition, fine motor, and language scores are positively correlated with each other suggest that improving one of these developmental domains may be beneficial to other domains. For example, activities promoting fine motor skills and encouraging children to express themselves vocally and verbally could potentially improve cognition in our study sample.

The very high stunting prevalence in the Vakinankaratra region combined with the low performance on child development assessments along with the observed limited resources for stimulation requires urgent attention. Similar results of low mean LAZ and low scores for several developmental domains including gross and fine motor, problem solving, communications, and socio-emotional development were reported in a randomized trial in southeastern Madagascar [37]. Combining efforts to prevent both undernutrition and developmental delay during the first 2 years in children has received much interest [20, 38]. However, results from integrated child nutrition and psychosocial stimulation interventions showed that improvements in growth do not always lead to better developmental outcomes, particularly as projects are scaled up [37, 39]. A recent cluster randomized trial in southeastern Madagascar showed no effect on developmental outcomes of an intensive counseling package on complementary feeding and child stimulation [37].

Nevertheless, the opportunity to address growth faltering and to promote optimal development during the early years should not be missed, especially in areas with high burdens of child undernutrition. The Nurturing Care framework suggested by the most recent Lancet series on Early Child Development integrates maternal and child health and nutrition as well as aspects of child protection, safety, and early learning opportunities [4]. Most previous interventions combined only improved nutrition and child stimulation components, but broadening the scope of action may be more effective [40]. For example, a parenting intervention consisting of peer group discussions and interactive learning activities on general child care (nutrition, hygiene, stimulation, and love) along with maternal psychological well-being in Uganda had positive effects on child development [41]. Also, using multiple learning channels such as educational materials, demonstrations, and problem-solving activities to increase adult provision of appropriate stimulation for young children may be more effective than using a single technique [42]. Conducting implementation research on the Nurturing Care framework in areas such as the Vakinankaratra region could provide valuable insights on how best to link various sectors to promote child development [43].

Although this study is the first, to our knowledge, to assess early child development in the Vakinankaratra region, our results should be interpreted with caution. First, although the Bayley Scales have been widely used to assess child development across different populations, the scale has not been validated in the Malagasy population. Validating the Bayley test was beyond the scope of this study. Additionally, we only assessed children from 11 to 13 months of age. Although different age groups may perform differently in child development assessments, previous studies reported low scores in language and cognitive skills among Malagasy children older than 2 years [21, 22]. Also, recall and social desirability bias are possible for the socioemotional assessment. Furthermore, mothers may not necessarily think much about all the questions pertaining to children’s behavior that were included in the questionnaire.

Despite the relatively low reported scores and the fact that two of the major risk factors for impaired child development, high stunting levels and widespread poverty, are present in the Vakinankaratra region, there is an urgent need to confirm or refute these results with a standardized Bayley assessment among Malagasy children. A larger sample size with enough power to be representative of the Vakinankaratra region and other regions with very high levels of stunting, including Amoron’i Mania (54%), Matsiatra Ambony (53%), and Bongolava (52%), would be helpful. With the appropriate sample size, in-depth analyses could be conducted to identify factors associated with each child development domain to complement this study. These additional data and information will allow the provision of recommendations for the support of child development in similar contexts as in central highlands of Madagascar. Alternatively, other child development assessment tools that could be easily adapted and integrated into large population-based studies, for example the INTER-NDA [44], are worthwhile to implement in contexts such as the central highlands of Madagascar.

## Conclusion

Cognition, motor, language, and socioemotional development scores of the evaluated children in the Vakinankaratra region were very low. Parents recognized the importance of interacting with their children but reported lacking time to do so. Interventions implemented early in infancy, thus taking advantage of the rapid growth and the high neural plasticity during the early years, are critical to promote growth and development in settings like the Vakinankaratra region with high burdens of malnutrition.

## Data Availability

The datasets used and/or analyzed during the current study are available from the corresponding author on reasonable request.

## Abbreviations

CNA: Community Nutrition Agents
FCI: Family care indicators
FGD: Focus group discussion
KII: Key informant interview
LAZ: Length-for-age *z*-score
LMICs: Low- and middle-income countries
NGO: Non-governmental Organization
SD: Standard deviation
WAZ: Weight-for-age *z*-score
WHO: World Health Organization
WLZ: Weight-for-length *z*-score
